# Progressive Locomotor Ataxy

**Published:** 1870-12

**Authors:** 


### PROGRESSIVE LOCOMOTOR ATAXY.

Clinic by PROFESSOR DAVIS.

MERCY HOSPITAL, *November 10, 1870.*

The patient before you presented himself a few days ago, at the Dispensary, for treatment. The physician in attendance, Dr. Lyman Ware, finding it a case of unusual interest, has brought him in, for your inspection to-day.

Rather more than a year ago, the patient was attacked with a sensation of weight or bearing down in the left groin, and extending around to the lumbar region, especially troublesome when he walked or attempted to go up stairs. A short time after the commencement of these symptoms, he had a sudden attack of blindness.

The blindness was partially relieved in a short time, although the sight has never been fully restored; and the bearing-down sensation, with some twitching, jerking in the muscles, has continued, more or less, up to the present time. The course of the lower two-thirds of the spine, the region of the left groin, and the entire surface of the lower extremities, you notice, is very sensitive to the touch. The slightest touch at any of these points causes quite as much or more pain than is produced by more decided pressure, showing plainly that there is a mere morbid sensibility of the parts, and not an inflammation in them. As the patient entered the hall, you noticed that he was somewhat uncertain in his movements, and inclined to steady himself by the table, chairs, or whatever was within his reach. When we requested him, however, to walk a short distance before you by directing his course to a certain point and fixing his attention upon his movements, he controlled them sufficiently so that there was no apparent unsteadiness *in* his gait; but, had his attention been suddenly withdrawn, he would probably have reeled, and been inclined to fall. There is, evidently, no paralysis present here, but the difficulty is owing entirely to an impairment of the power of co-ordination.

The impairment in this case is so slight that the unsteadiness of the movements would not ordinarily be noticed without the attention was directed specially to them.

In cases further advanced, the gait becomes unsteady and reeling, as that of an intoxicated person. Special difficulty is experienced in mounting stairs on account of an inability to raise the feet from step to step. The patients frequently complain of acute pains of a variable character, like those of rheumatism, moving from one organ or portion of the body to another.

Perverted sensations, not actual pains, are also experienced, such as thrills and jerkings in the muscular strictures, like shocks from a galvanic battery. These alterations of sensibility are derived from the nerves of sensation, and are associated particularly with this condition of failing co-ordination.

Should pain in the head and dizziness be complained of among the prominent symptoms, some disease in the cerebrum or cerebellum should at once be suspected. When, however, the first and most prominent symptoms are a morbid sensibility of the extremities and impairment of co-ordination, we may be pretty sure that we have a case of progressive locomotor ataxia.

The progress of the disease is slow at different stages of its course; it may even remain stationary for considerable periods Its progress is always resumed, however, and generally continues to the end in spite of any course of treatment which has, as yet, been discovered.

Sudden attacks of blindness, either partial or total, almost always occur in connection with the disease. Frequently, dark spots are perceived, apparently floating before the eyes, and the pupils are largely dilated. Where opportunity has been offered for examination after death in these cases, the optic nerve has been found to be more or less atrophied. The principal lesion, however, consists of an alteration in the deposit of nerve structure in the lower third of the spinal cord. This alteration may be limited to a space of not more than two or three inches in length, and consists of a deficiency of nerve substance, and a substitution for it of amylaceous or fatty matter. The fibrous or connective tissue of the cord is not atrophied, but rather increased or thickened. This atrophy extends also to the roots of the spinal nerves.

The direct cause of the disease is involved in obscurity. All the cases that I have met with have occurred to persons addicted to the immoderate use of tobacco, and I am inclined to consider this a predisposing cause, although it cannot, of course, be considered a direct exciting cause. I think that the excessive use of tobacco retards nervous growth and nutrition. Other influences have also been assigned as predisposing causes, such as excessive sexual excitement, either solitary or social. It would be logical to suppose that everything which exhausts and impairs nervous nutrition, would act as a predisposing cause—a damp, changeable climate, and occupation in damp, unhealthy rooms, would be classed among these causes. It is probable, however, that there is something in the system of the patient which acts as the direct exciting cause.

Treatment.—As regards the possibility of checking the progress of the disease by any course of treatment, we must confess that experience has not given us the best encouragment to hope for favorable results. It used to be the custom to blister, cauterize, introduce seatons, etc. These measures were found, however, to only hasten the development of the disease. Alcoholic stimulants, recommended by some authorities, are also contra-indicated on account of their action in retarding healthy nutrition and promoting the tendency to fatty degeneration. What we wish to accomplish is, to bring back a healthy nutrition of the nerve structures. Phosphorous is well known to be an abundant element in nerve structures, and we may expect, therefore, to derive some benefit from the administration of phosphoric acid and the phosphites—generally prescribe phosphoric acid, combined with compound syrup of the hypophosphites; a teaspoonful before each meal. To lessen the morbid sensations, think that fluid ext. cannabis indica, 10 to 15 drops, three times a-day, is the most appropriate in those cases. The opiates produce too much general derangement. Belladonna, Hyosciamus, and ergot, all tend to produce the quieting effect desired. The ergot is liable, however, to produce too much capillary contraction. If digestion is not good, should prescribe liquid Rennett, teaspoonful after each meal.

The patient should be allowed a good, plain, substantial diet, plenty of milk, milk-whey, buttermilk, etc. No tea or coffee should be allowed, or at least only a single cup, very weak.

Alcoholic stimulants and tobacco should be rigidly excluded. The patient's chances of recovery will be greatly increased, if he will leave off the use of tobacco entirely.

The patient should be encouraged to take a moderate amount of exercise in the open air every day, but not enough to produce excessive weariness; must be careful not to overtax the strength. Such a course, if followed out, offers a better prospect of benefit than any other.

